# Cobbler’s Awl Causing a Rare Pediatric Paraspinal Injury Managed Using 3D CT

**DOI:** 10.7759/cureus.8942

**Published:** 2020-07-01

**Authors:** Jojo James, Mahesh Machavarapu

**Affiliations:** 1 Surgery, Tata Main Hospital, Jamshedpur, IND; 2 General Surgery, Tata Main Hospital, Jamshedpur, IND

**Keywords:** paediatric, paraspinal, penetrating, cobblers awl, 3d ct scan

## Abstract

Pediatric spinal injuries are very uncommon, accounting for a small percentage of all spinal injuries. Domestic accidents such as falling and bumping are frequent events during childhood. In this case report, we present a rare penetrating trauma by a cobbler’s awl at the paraspinal level. The patient was referred to the ED after a needle became impaled into his back due to an accident that occurred at home. The patient’s neurologic assessment was normal. A radiologic study of the patient showed a cobbler’s awl penetrating the paravertebral muscle at the fourth lumbar vertebra level. The needle was removed promptly after an emergency surgical procedure. Postprocedure no complications occurred.

## Introduction

Pediatric spinal injuries are very rare conditions and account for 1%-10% of all spinal injuries [1]. Children often get involved in falls and trips and thereon suffer from injures of varying severity, some requiring surgical intervention. Amongst these, penetrating injuries involving the spinal and paraspinal area secondary to sharp devices are rare. The case of a young boy is presented here, in whom a cobbler’s awl pierced the paraspinal region during a domestic household accident. Our case is unique in the unusual location and the instrument causing such a mode of injury and to our knowledge has not been reported till date.

## Case presentation

**Figure 1:**
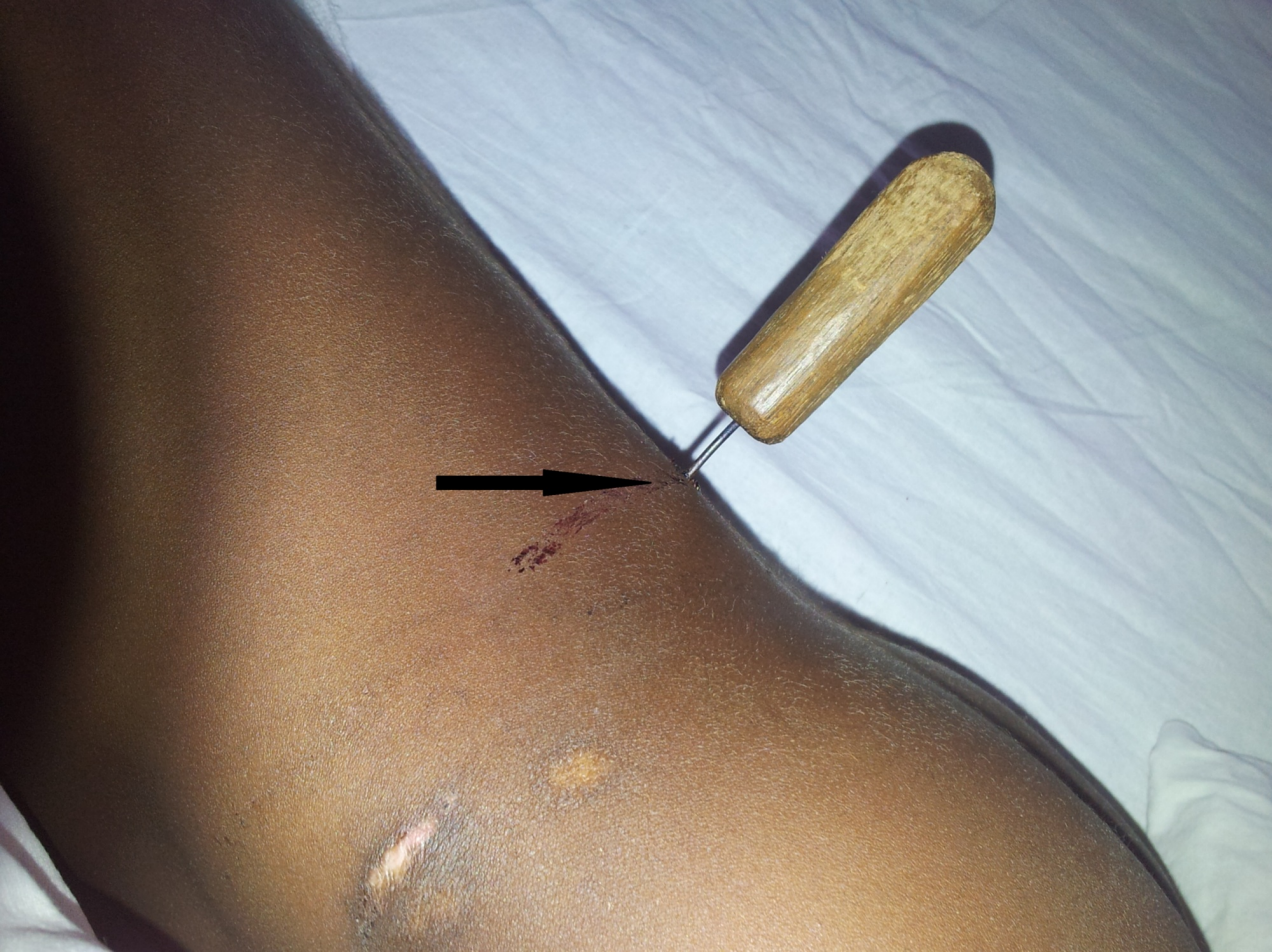
Patient lying with cobbler's awl impaled in the back.

A seven-year-old male child of a local cobbler presented to the ED with a history of an accidental impalement with a cobbler’s awl. He was transported in a lateral position with the awl in situ. The child was conscious and oriented with vital parameters recorded at a pulse rate of 110 per minute and blood pressure of 100/60 mmHg. On examination, the awl was seen impaled on the left of the spine at the level of fourth lumbar vertebra (L4), 3 cm above the left sacral bone (Figure 1).

**Figure 2:**
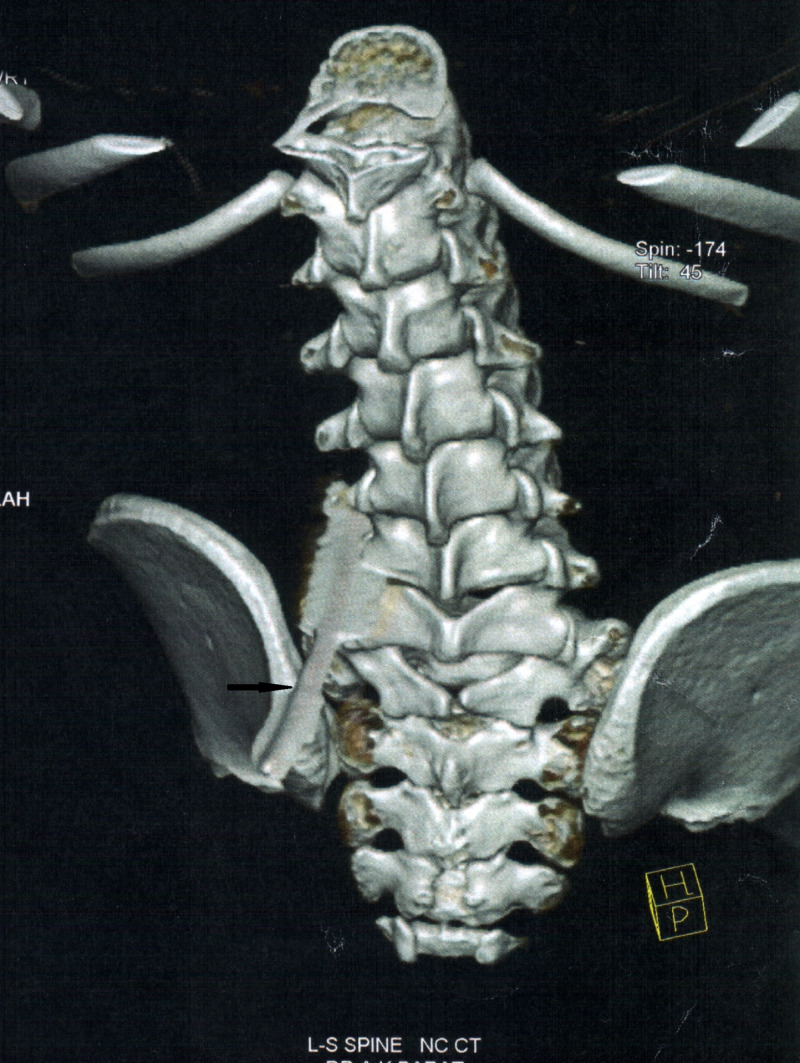
3D reconstruction posterior view showing entry point of cobbler's awl at the L4 level.

A neurologic examination of his limbs showed a complete range of movement with normal strength with normal anal tone and contraction. His abdomen was soft and he voluntarily passed clear urine. He was put on systemic antibiotics and once stable was taken up for a CT scan with 3D reconstruction to visualize and plan the appropriate surgical intervention. The scan revealed that the awl missed the spine and was lodged in the soft tissue around the lumbar vertebra at the L4 level (Figure 2).

**Figure 3:**
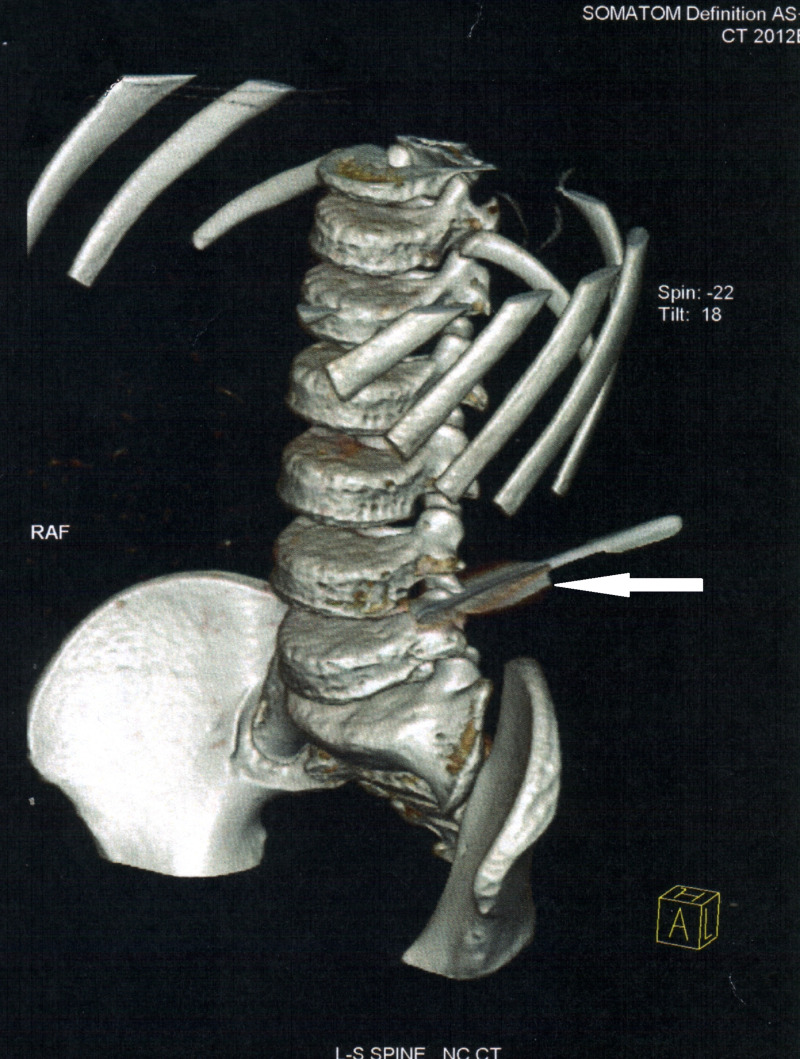
3D reconstruction lateral view showing entire track of cobbler’s awl.

The lateral view demonstrated that the path of awl was between L3 and L4 vertebra and the direction and depth of penetration were deflected off the body of the fourth lumbar vertebra. There was no obvious injury noted to the surrounding structures (Figure 3).

He was posted for surgery for removal of the awl under general anesthesia and the awl was removed cautiously after which there was no active bleeding or discharge. The wound was washed copiously with warm saline. The postoperative period was uneventful and after a period of three days, a neurologic examination ensured he had no neurologic deterioration and was discharged.

## Discussion

Spine injuries are rare in children. Osenbach and Menezes studied childhood spinal trauma in 179 children and found that cervical trauma (63%) was the most frequently encountered condition, while thoracic (13%), thoracolumbar (11%), and trauma to the lumbar region (14%) were rare [2]. The etiology of pediatric injuries differed from that of adult injuries in that falls were the most common causative factor (56%) followed by vehicular accidents (23%) [3].

We wish to report our case to highlight three aspects which as per our knowledge are unusual. Firstly, the uniqueness of the implementation of injury. Trauma due to penetrating foreign objects to the spine is rare. The instruments of such injuries include knives, wooden materials, glass, pencils, firearms, screw driver, and described herein, a cobbler’s awl has not been reported before.

Secondly to highlight the behavior, clinical approach, and sequelae of such trauma in a pediatric group. The spinal column of children is different from that of the adult. As children grow, the ossification centers enlarge leading to reversal of cartilage/bone ratio [4]. This is also thought to be the reason why the neurologic recovery in children with spinal cord injuries is thought to be better than that in the adult population, this being adequately demonstrated in our patient. Penetrating spinal trauma in children also differs from the therapeutic approach applied to adults. A child may not be able to express pain and sensitivity, and therefore a complete systemic examination must be conducted and local dermal lesions and injuries detected.

In a pediatric age, it maybe difficult to determine the site of entrance of a foreign object as this relies heavily on the history which in this group may not be very accurate. Hence our third point emphasizes on the role of appropriate diagnostics in managing such situations effectively. The CT scan can illuminate the trail of the injury and help in the safe removal of the foreign body during surgery. The 3D reconstruction of plain CT images gave us added advantage in assessing the depth and path of injury caused by the awl and ruled out the possibility of other organ injuries or complication. A broken piece of foreign body may present later with neurologic symptoms, hence it is imperative not to miss such injuries before surgery [5-6]. Metal artifact may obscure some images, however, bone density images show the relationship between the metallic object, the spinal cord, and bony fragments [7]. Preoperative magnetic resonance (MR) is not recommended in such cases due to the risk of movement by the strong magnetic field that in turn may worsen the neurologic deficit [8].

Infections originating from the normal dermal flora may complicate such injuries, hence prophylactic antibiotic therapy must be started against these bacteria [9]. In our case, ceftriaxone was used as prophylactic antibiotics and no infections developed. Anatomically direct central backstabbings rarely produce injuries to the spinal cord and central retroperitoneal structures due to the protection provided by the layers of muscle and the spinal column, with the spinous and transverse processes deflecting blades laterally [10]. No immediate or delayed complications developed in our patient.

## Conclusions

Trauma due to penetrating foreign objects in the pediatric spinal region is important because of the location, and therefore early surgical intervention should be considered. The use of 3D reconstruction CT images to assess the path of penetrating foreign objects, the penetrated tissue, the site and the damage caused help to precisely dictate the nature of the surgery and its outcome. Managing pediatric spinal injuries is a challenge, as the clinical approach and the recovery process are different and using 3D CT scan reconstruction helped in our case and we wish to highlight these aspects by our case report.
